# Genetic contribution of breast cancer genes in women of black African origin

**DOI:** 10.3389/fgene.2023.1302645

**Published:** 2023-12-13

**Authors:** Rokhaya Ndiaye, Jean Pascal Demba Diop, Ahmadou Dem, Alioune Dieye

**Affiliations:** ^1^ Division of Human Genetics, Faculty of Medicine, Pharmacy and Odonto-Stomatology, University Cheikh Anta DIOP, Dakar, Senegal; ^2^ Department of Oncology, Faculty of Medicine, Pharmacy and Odonto-Stomatology, University Cheikh Anta DIOP, Dakar, Senegal; ^3^ Department of Immunology, Faculty of Medicine, Pharmacy and Odonto-Stomatology, University Cheikh Anta DIOP, Dakar, Senegal

**Keywords:** breast cancer, genetic contribution, genes, black African origin, women

## Abstract

Breast cancer (BC) is an increasing public health issue worldwide. BC incidence and mortality rates are rising in transitioning countries in Africa, with the most rapid increase occurring in Sub-Saharan Africa (SSA). Female BC represents 25.8% of all cancer diagnosis in SSA. Early age at onset, high grade and triple negative tumors are hallmarks of BC in this region, associated with germline pathogenic variants in susceptibility genes. While several genes have been associated with genetic predisposition (BRCA1, BRCA2, PALB2, TP53, PTEN, CDH1, STK11, ATM, CHEK2, NBN, BARD1, BRIP1, RAD50, RAD51C, RAD51D, … ), most studies have reported contribution of BRCA1 and BRCA2 pathogenic variants. Genetic contribution of BRCA genes has been estimated at 27% in Caucasian women. Available data from population of African origin are scarce and have mainly focused on pathogenic variants of BRCA1 and BRCA2. Reports from main studies on large sample size highlighted that BRCA1 still the major gene associated with BC in SSA. In addition, BRCA2, PALB2, and P53, are also on the top major genes with high penetrance, associated with BC. Mutation spectrum of BC genes in black African women seems to be different from Caucasian with increasing number of founder mutations identified. We hypothesis that the genetic contribution of known BC genes may be different between women of black African origin compared to Caucasians. In this review we explore the genetic contribution of known breast cancer genes in women of African origin, and discuss perspectives for prevention and patients care strategies in the era of precision medicine.

## 1 Introduction

Breast cancer is the most commonly diagnosed cancer and the second leading cause of cancer death in African population. Despite having a lower breast cancer incidence rate due to lack of cancer registries, African population show significantly higher mortality rate of breast cancer (DeSantis et al., 2019). Genetic factors contribute significantly to the aetiology of breast cancer (Diaz-Zabala et al., 2022). Recently notable advances have been reported in knowledge of both prevalence and penetrance of germline inactivating mutations in genes that are associated with a moderate or high risk of breast cancer. To date, 12 breast cancer predisposition genes including *ATM*, *BARD1*, *BRCA1*, *BRCA2*, *CDH1*, *CHEK2*, *NF1*, *PALB2*, *PTEN*, *RAD51C*, *RAD51D*, *and TP53* have been identified (Tischkowitz et al., 2012; Antoniou et al., 2014; Easton et al., 2015; Couch et al., 2017; Palmer et al., 2020). Additional genes including *CDKNA2A*, *MRE11A*, *MSH1*, *MSH*2, *MSH6*, and *PSM2* have been linked to breast cancer risk, although their etiologic roles have not yet been well established. Compared to studies conducted in women of Caucasian ancestry, studies in black African women have much smaller sample sizes and have focused mainly on mutation screening of *BRCA1*, *BRCA2* (Nanda et al., 2005; Pal et al., 2015; Zheng et al., 2018). It has been shown that African women have an increased likelihood of being diagnosed at younger age, with Triple Negative and high grade tumours, suggesting an ancestral or hereditary component. Meanwhile this population is significantly less likely to be referred for genetic testing due to lack of local infrastructures (Levy et al., 2011; Cragun et al., 2017). Very few studies have focused on mutational spectrum of breast cancer genes in African women. In this review we summarize the genetic contribution of known breast cancer genes in black women of African origin, and discuss perspectives for prevention and patients care strategies, in the era of precision medicine.

## 2 Genetic contribution of breast cancer genes in black African women

Since the identification of the first BC predisposition gene in 1994 (Miki et al., 1994), almost 11 genes with high or moderate penetrance have been identified (Palmer et al., 2020). The involvement of these genes and their potential risks have been exclusively documented in women of Caucasian origin. For black African women very few studies aiming to identify mutational spectrum of all breast cancer susceptibility genes in a large set of population, have been carried out. Reported studies are from Ghana, Nigeria, Cameroon and Uganda (Pal et al., 2015; Cragun et al., 2017; Ahearn et al., 2022). Other reported studies focused on *BRCA1* and *BRCA2* genes in limited families or sporadic cases. This is the case for South Africa, Senegal, Burkina Faso and Mauritania (Levy et al., 2011; Diop et al., 2021; Ouedraogo et al., 2022; Van der Merwe et al., 2022) (Table 1).

**TABLE 1:** Pathogenic variant frequencies of breast cancer major genes in black African and Caucasian women.

Country	Nigeria (10)	Cameroon/Uganda (18)	Ghana (14)	Brazil (22)	United States (African-American) (7)	Europe and Asia (24)
Population ancestry	African			African origin		Caucasian
Population size	1,136	196	871	173	4,993	48,826
Mutation rate	14.7% (167/1,136)	15.8% (31/196)	12.7% (111/871)	20.8% (36/173)	8.23% (416/5,054)	9.7% (4,754/48,826)
*BRCA1*	7% (80)	5.6% (11)	2.64% (23)	6 .9% (12)	1.6% (81)	1.05% (515)
*BRCA2*	4.1% (47)	5.6% (11)	3.78% (33)	5.8% (10)	1.94% (98)	1.54% (754)
*PALB2*	1% (11)	1.5% (2)	0.80% (7)	1.7% (3)	1.05% (53)	0.56% (274)
*TP53*	0.4% (5)	1% (1)	0.57% (5)	0.6% (1)	0.10% (5)	0.01% (7)
Others genes	2.1% (24)	3.05% (6)	5.39% (47)	6.4% (11)	3.65% (179)	6.56% (3,204)

In Nigeria a study including 1,136 women with invasive BC and 997 controls by Zheng et al. (2018), reported that among patients with breast cancer, 14.7% carried a pathogenic or likely-pathogenic mutation in breast cancer genes, whereas in controls, 1.8% have mutations. *BRCA1* was the first gene with the highest contribution (47.9%). The other genes with subsequent high risk were *BRCA2* (28.1%), *PALB2* (6.5%), and *TP53* (2.9%). Ten other genes *ATM, BARD1*, *BRIP1*, *CHEK1*, *CHEK2*, *GEN1*, *NBN*, *RAD51*, and *XRCC2* were reported with a pathogenic variant in one or few patients (Zheng et al., 2018).

In Ghana a 34 genes panel from BRIDGES have been used to sequence 871 breast cancer cases and 1,563 controls. Ahearn et al. (2022) reported that 12.7% of patient carried a pathogenic variant and 1.22% for control population in studied genes. Pathogenic variant in *BRCA2* gene conferred the highest risk (29.7%) followed by *BRCA1* (20.7%). *PALB2*, *ABRAXAS1*, *FANCC*, and *TP53* were also associated with significant increase in BC risk, with 9 patients for *PALB2*, 7 for *ABRAXAS1*, 5 patients for *FANCC*, *TP53*. Fifteen others genes *ATM*, *BABAM2*, *BARD1*, *ATM*, *CHEK2*, *EPCAM*, *GEM*, *MSH2*, *MUTYH*, *NBN*, *PMS2*, *PTEN*, *RAD50*, *RAD50*, *RAD51C*, *RAD51D*, *RINT1*, *PTEN* also harboured pathogenic variant in one or few patients (Ahearn et al., 2022).

For women from Uganda and Cameroon, screening of the same 34 genes panel reported 15.8% pathogenic variant frequency for recruited patients. With a pathogenic variant frequency of 47% identified, BRCA1 was the most mutated gene in Cameroonian women while in Uganda *BRCA2* (50%) was the most mutated gene. Other genes such as *ATM* (8.5%), *PALB2* (5.7%), *TP53* (2.8%), *CHEK2*, *BARD1*, *CDH1* also carried pathogenic variant for one or few women (Adedokun et al., 2020). For control population 3 had pathogenic variant in BRCA1 (1.1%) and BARD1 (0.5%).

Studies implemented in other African countries targeted limited families or sporadic BC cases. Most of them focused on the major genes *BRCA1* and *BRCA2*, and highlighted strong genetic contribution of *BRCA1* c.814_824dup10 in Senegal (56%) and Mauritania (34.2%). The variant was reported with high allelic frequency, respectively 30% and 4.7% in Senegal and Mauritania with a founder effect (Diop et al., 2021; Brahim et al., 2022). In South Africa *BRCA2* gene had the highest contribution (11.8%) with identification of 2 black founder variants *BRCA2* c.582G>A and *BRCA2* c.5771_5774del. Oosthuizen et al. (2020) Data from Burkina Faso did not show strong genetic contribution of *BRCA* genes (Table 2).

**TABLE 2:** BRCA genes contribution in other Sub Saharan African countries.

Countries	Senegal (15)	Mauritania (19)	Burkina Faso (16)	South Africa (17)
Population size/type	46/families	132/families and sporadic cases	133/sporadic cases	2,894/families and sporadic cases
*BRCA1*	56.5% (26)	19.6% (26)	0.75% (1)	4.9% (143)
*BRCA2*	2.1% (1)	9.09% (12)	1.5% (2)	11.81% (342)

In light of these studies, it appears that genetic contribution to BC is heterogeneous between SSA countries and accounts for 12.7%–56% of BC cases (Diop et al., 2021; Ahearn et al., 2022). The 4 major genes contributing to BC are *BRCA1*, *BRCA2*, *PALB2*, and *TP53* while population specific major genes have been raised. These results also highlighted the variability of the genetic contribution of BC genes through the African continent, reflecting the high genetic diversity observed in African populations.

## 3 Genetic contribution of breast cancer genes in women of black African descent from the diaspora

Women of African descent across the diaspora, as native black African women, have the worst outcomes from breast cancer compared to Caucasian women. It has been shown that African-American women have higher incidence of breast cancer at young age, higher incidence of Triple Negative Breast Cancer (TNBC) and higher breast cancer mortality rate (42%) than non-Hispanic white Women (Dunn et al., 2010; DeSantis et al., 2019). Palmer et al. (2020) reported in 2020, pathogenic variants in a panel 23 genes tested in 416 women out of 5,054 African-American women diagnosed with BC (8.2%) and 114 out of 4,993 unaffected women (2.2%). Among BC patients, 1.6% (81) had *BRCA1* mutations, 1.9% (98) in *BRCA2* and 1% (53) in *PALB2*. Mutations in other genes including *CDH1*, *NF1*, *PTEN*, and *TP53* were observed in 15 affected women. For the 12 BC known genes conferring moderate or high risk in women of European Ancestry (*ATM*, *BARD1*, *BRCA1*, *BRCA2*, *CDH1*, *CHEK2*, *NF1*, *PALB2*, *PTEN*, *RAD51C*, *RAD51D*, *and TP53*), mutations were identified in 6.5% of affected women (Palmer et al., 2020). Moreover, a recent study by Diaz-Zabala et al. (2022) showed a higher frequency of 7.33% (Table1).

Although, given the high prevalence of aggressive BC reported in young Brazilian women, GES et al. highlighted in a cohort of black women of African slaves descent, 24.1% (28/116) carrying pathogenic variants in *ATM, BARD1, BRCA1*, *BRCA2*, *BRIP1*, *FAM174A*, *FANCM*, *PALB2*, *and TP53*. The highest contribution was observed for *BRCA1* (32%) followed by *BRCA2* (27%) (Felix et al., 2022) (Table1).

## 4 Genetic contribution of breast cancer genes in black women compared to Caucasian women

**FIGURE 1:**
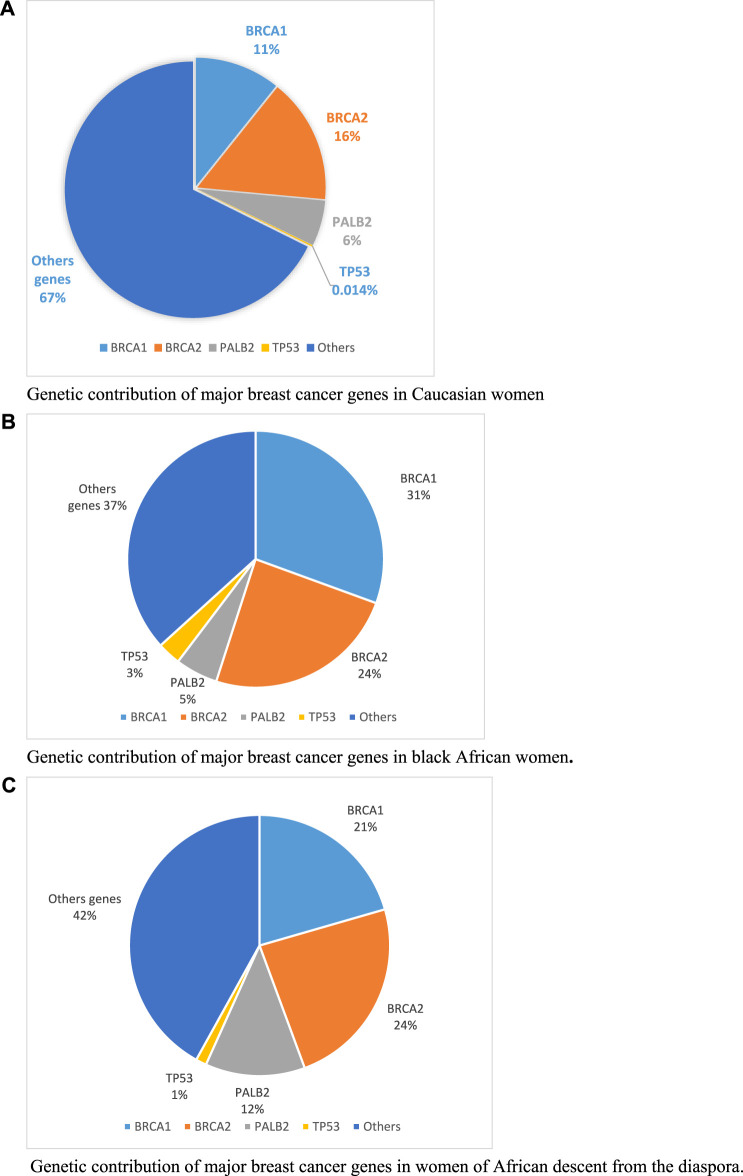
Genetic contribution of major breast cancer genes in Caucasian, black African and women of African descent from the diaspora. **(A)** Genetic contribution of major breast cancer genes in Caucasian women. **(B)** Genetic contribution of major breast cancer genes in black African women. **(C)** Genetic contribution of major breast cancer genes in women of African descent from the diaspora.

Genetic contribution of *BRCA* genes has been estimated at 9.7% in Caucasian population in 2014 (Couch et al., 2014). More recently, in a larger study on 113,000 Caucasian women, the genetic contribution of *BRCA* genes was estimated at 27% (Dorling et al., 2021) (Figure 1A). This is lower compare to native black African women or black women from the African diaspora where the overall genetic contributions of *BRCA* genes from reported studies, are estimated at 55% and 45% respectively (Figures 1B, C). This could be explained by the high consanguinity rate observed in African population leading to higher prevalence of founder mutations as observed in SSA countries.

Beside *BRCA* genes, *PALB2* and TP53 are the second BC contributors for native black African women. While *PALB2* has similar contribution in both native black African and Caucasian women, *TP53* only contributes in black African women (1%–3%) compared to Caucasian (0.014%). This observed variability on BC genes contribution across populations highlights the need to investigate the complete molecular characterisation of breast cancer in SSA in larger patient cohorts. This will certainly open insights into new therapeutic strategies and personalized approaches for better patients’ care.

## 5 Future perspectives

Despite being the most genetically diverse continent, African populations still under-represented in genomic databases. It is urgent for African countries to characterize the genetic architecture of their populations but also identify specificities related to molecular profiles of diseases in African patients. In this review we showed that scarce studies have been implemented to dissect the contribution of known genes associated with predisposition to breast cancer in black African women. This is due to unavailability of genetic testing infrastructures and sequencing platforms throughout the continent. The high contribution of *BRCA1* and *BRCA2* genes in SSA women suggested the need to urgently implement low cost genetic testing and also genetic counselling services to improve BC patients’ care. *BRCA* testing is usually offered in clinical practice to allow more choices for therapeutic and prevention strategies. Currently two PARP inhibitors, olaparib and talazoparib, have been approved by the United States Food and Drug Administration (FDA) and European Medicines Agency (EMA) for BC patients with deleterious or suspected deleterious *BRCA* mutations. These targeted therapies are not affordable nor available for BC African patients. It is therefore an urgent need to fully characterize BC genetic profile in each country and translate these results into more efficient and resilient therapies for African patient.

## 6 Conclusion

In this review we have highlighted that *BRCA* and *PALB2* genes are major contributors in breast cancer predisposition in black African women and Caucasian. Meanwhile, the genetic contribution of *BRCA1* and *BRCA2* seems to be much higher in African women. Since very few studies with large sample size have been implemented in black African BC women, it is of high interest to dig into this gap by characterizing the full genetic profile of breast cancer by whole Exome or whole Genome sequencing. New therapeutic perspectives will then be opened to reduce the breast cancer burden in Africa.
